# Spatiotemporal Response of Hydrological Drought to Meteorological Drought on Multi-Time Scales Concerning Endorheic Basin

**DOI:** 10.3390/ijerph18179074

**Published:** 2021-08-27

**Authors:** Nina Zhu, Jianhua Xu, Gang Zeng, Xianzhong Cao

**Affiliations:** 1 The Center for Modern Chinese City Studies, Institute of Urban Development, East China Normal University, Shanghai 200062, China; ninaecnu@126.com (N.Z.); xzcao@geo.ecnu.edu.cn (X.C.); 2 Key Laboratory of Geographic Information Science (Ministry of Education), East China Normal University, Shanghai 200241, China; 3 Research Center for East–West Cooperation in China, East China Normal University, Shanghai 200241, China

**Keywords:** standardized precipitation index, standardized terrestrial water storage index, spatiotemporal changes, multi-time scale response, endorheic basin

## Abstract

Under the controversial background of “Northwestern China is gradually developing towards warm and humid”, how hydrological drought responds to meteorological drought at the endorheic basin is of great significance. To address this problem, we first analyzed the spatiotemporal variation of meteorological and hydrological droughts at Tarim Basin River from 1960 to 2014 by using the daily standardized precipitation index (SPI) and daily standardized terrestrial water storage index (SWSI) based on the reanalysis data. Thereafter, we explored the spatiotemporal response of hydrological drought to meteorological drought on the multi-time scale by using the cross-wavelet transform method, Ensemble Empirical Mode Decomposition (EEMD), and correlation analysis. We find that: (1) both meteorological and hydrological droughts signified a gradually weakened trend in time; (2) meteorological and hydrological drought have significant resonance periods on the 10-month time scale and the 8-year time scale; (3) hydrological drought generally lags behind the meteorological drought by 7 days in plains areas, while it can last as long as several months or even a year in mountainous areas.

## 1. Introduction

Drought is a common natural phenomenon worldwide [1]. Since ancient times, both the economy and society of mankind have been affected by droughts [2]. The most direct impact is the reduction in food production and difficulties in obtaining clean water [2]. Unfortunately, severe drought will lead to the death of humans and livestock, and ultimately the demise of civilization [3]. In the past millennium, most countries and regions have suffered from drought [3]. For instance, the economic losses caused by droughts in the United States average 6–8 billion dollars per year. From 1950 to 2017 in China, the average disaster area attributed to droughts was 205,021.7 km^2^, and the average food loss was 16.26 million tons. In Africa, during the 1980s, more than half a million people died due to droughts. In total, worldwide losses due to droughts accounted for 42% of total natural disaster losses [4]. Additionally, with an increase in global warming and the intensity of human activity, the original water balance is being disrupted, making the climate-hydrological system more fragile and complex. In turn, various extreme climatic events, such as drought and waterlogging, frequently occurred with more severe consequences [5]. Currently, the issue of droughts has been prioritized by various countries and regions [6].

In China, and especially the Tarim River Basin of northwest China, the issue of droughts is a major problem. As an endorheic basin, this area is indispensable for the ancient Silk Road and the One Belt and One Road project, and it is an important bridge connecting China to the European countries. Simultaneously, the region has an abundance of natural resources and is reserved to cater to China’s energy demands. The importance of this region is self-evident, however, the Tarim River Basin has always been facing severe drought problems, which has brought great challenges to local development and domestic water utilization. The geographical environment and regional climatic process of the Tarim River Basin are complex [7], with mountains, deserts, and oases and rare precipitation, strong evaporation, large temperature difference, and long hours of sunshine [6,8]. The high mountains produce runoff whereas the desert dissipates it. Besides, the inland river basins in northwest China are vast, while the measured meteorological and hydrological data are scarce. Thus, it is difficult to quantitatively assess the variation of droughts in the Tarim Basin River, especially the spatial and temporal response of hydrological drought to meteorological drought.

There are both differences and connections between meteorological and hydrological droughts, and they do not occur at the same time. Generally, when a meteorological drought persists for some time, a hydrological drought will gradually form. Accordingly, the hydrological drought lags behind the meteorological drought [9,10]. To explore this phenomenon, many scholars have conducted extensive research encompassing drought duration, drought intensity, and drought severity via statistical methods or numerical model simulation methods [11,12,13,14,15]. Zhao et al. [16] investigated the variations in hydrological and meteorological droughts and the response of hydrological drought to meteorological drought at the Jing River of northwest China, revealing that the hydrological drought lagged behind meteorological drought by 127 days. Moreover, Huang et al. [11] inspected the response of hydrological drought to meteorological drought at Wei River by using standardized precipitation index (SPI) and standardized runoff index (SRI), and the result validated that the response time had obvious seasonal differences: the response time of spring and summer was shorter than that of autumn and winter. Ma et al. [10] distinguished the lag time of hydrological drought in relation to meteorological drought through the correlation between the two and separated the contribution rate of human activities and climate change, according to the lag time. Likewise, scholars have administered various studies on the response of the hydrological drought to the meteorological drought at the Tarim River Basin [17,18,19]. However, due to the deficiency of meteorological and hydrological data regarding the Tarim River Basin, scholars have mostly targeted the temporal response of hydrological drought to meteorological drought without determining the spatial response.

It was for the above reasons that we selected the Tarim River Basin as a typical basin for this study. Based on the reanalysis data from 1960–2014, we first used indicators of SPI and surface water supply index (SWSI) to define the spatiotemporal patterns of meteorological and hydrological droughts. Afterward, we discerned the temporal response and then the spatial response of hydrological drought to meteorological drought on multi-time scales.

## 2. Materials and Methods

### 2.1. Study Area and Data

#### 2.1.1. Study Area

**Figure 1:**
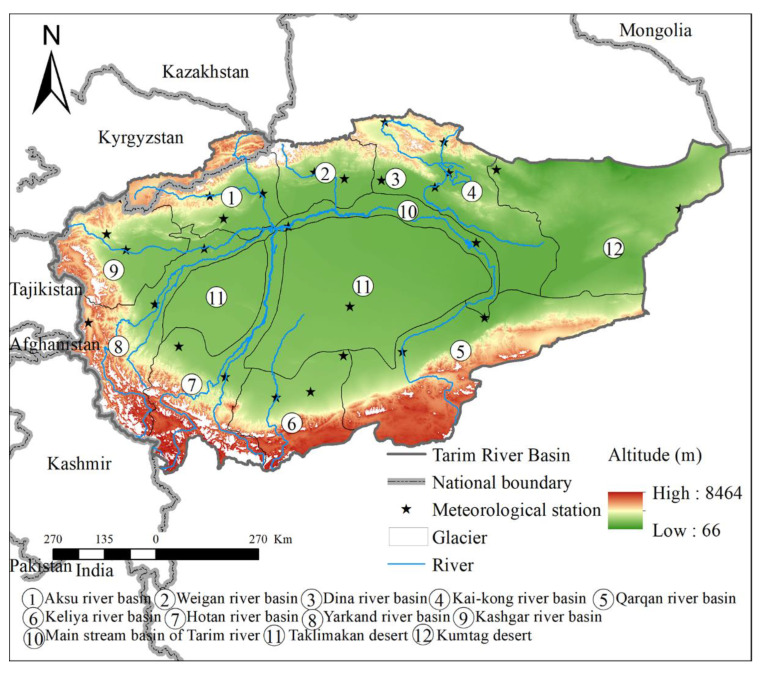
Location of the Tarim River Basin.

The Tarim River Basin is an endorheic basin and is located in the southern part of Xinjiang, between the Tianshan Mountains and the Kunlun Mountains of China. Its range is 73°10′ E–94°05′ E and 34°55′ N–43°08′ N. The topography of the Tarim River Basin is complex, with high mountains, plains, and deserts distributed across the basin. Specifically, the mountainous, plain, and desert areas occupy 47%, 20%, and 33% of the landmass, respectively [14]. Primarily, the entire basin is split by the Tarim River, which constitutes nine major river systems, namely the Aksu, Weigan, Dina, Kai-kong, Qarqan, Keliya, Hotan, Yarkand, and Kashgar rivers. These nine rivers span over nine major basins, which together with the main Tarim River Basin constitute the entire Tarim River Basin. Figure 1 also contains the Taklimakan desert and Kumtag desert, as illustrated below. From Figure 1, we can see that the Tarim River Basin borders Mongolia, Kazakhstan, Kyrgyztan, Tajikistan, Afghamstan, and Kashmir. The altitude of the Tarim River Basin is between 66 m and 8464 m, and there are a large number of glaciers in the mountains, especially in the mountains in the southern part of the basin. The river in Figure 1 represents the Tarim River, which runs through the entire Tarim River Basin. According to the distribution of tributaries, the entire Tarim River Basin is divided into different sub-basins. There are 12 sub-basins in Figure 1, namely Aksu River Basin, Weigan River Basin, Dina River Basin, Kai-kong River Basin, Qarqan River Basin, Keliya River Basin, Hotan River Basin, Yarkand River Basin, Kashgar River Basin, Main Stream Basin of Tarim River, Taklimakan Desert, and Kumtag Desert. In addition, there are 26 meteorological stations scattered in various sub-basins.

The Tarim River Basin belongs to a temperate continental climate, where precipitation is rare, with an average annual precipitation of only 116.8 mm, while evaporation can reach 24 times the precipitation [20]. Besides, the precipitation is not evenly distributed in space, as it is reported to be more in mountainous regions as opposed to plains and deserts. The average annual precipitation can reach up to 500 mm or more in the Tianshan mountain area, while the average annual precipitation is less than 50 mm in the desert area. From the southeast to the northwest, the precipitation of the whole basin indicates an increasing trend. In recent decades, the precipitation in this basin has shown a gradually increasing trend due to climate change, and the increment of precipitation in mountain areas is more than in plain areas [19]. The unique geographical location and the nature of the underlying surface causes the Tarim River Basin to have large annual and daily temperatures. It is the hottest in July, with an average monthly temperature of 20–30 °C, and the coldest in January, with an average monthly temperature between −20 to −10 °C. The temperature in this basin also presents an increasing trend with global warming, and the rate of increase in the plains areas is higher than that in mountainous areas [20]. The characteristics of high altitude, high latitude, and low precipitation make the photothermal resources here very rich. The annual average sunshine hours are 2847 h. Notably, the longest daily sunshine hours can reach 14.9 h, and the annual average solar radiation is 618 kJ/cm^2^. The solar thermal resources are conducive to the accumulation of sugar, making it a famous place for melons and fruits. Moreover, the crops here are mainly cooked twice a year.

#### 2.1.2. Data

To explore the response of hydrological drought to meteorological drought, daily precipitation (mm) and daily terrestrial water storage (kg/m^2^) are used. They are the Global Land Data Assimilation System (GLDAS) data provided by the National Aeronautics and Space Administration (NASA) (http://disc.gsfc.nasa.gov) (accessed on 23 May 2020). These data are part of the reanalysis data. The reanalysis data refer to the use of advanced global data assimilation systems and complete databases to perform quality control and the assimilation of observational data from various sources (ground, ship, radiosonde, wind balloon, aircraft, satellite, etc.) to obtain a complete set of reanalysis datasets. The reanalysis data not only contain many elements and a wide range but also extends over a long period of time, which is a comprehensive dataset. The GLDAS dataset mainly uses advanced data assimilation and surface modeling technology to extract satellite data and ground observation data to generate the best land surface state and flux field. Presently, GLDAS has four types of land surface models: Noah, Catchment, the Community Land Model, and the Variable Infiltration Capacity, which contain two versions of data, GLDAS-1 and GLDAS-2. Specifically, GLDAS-2 is an improvement in GLDAS-1. Furthermore, the spatial resolution of GLDAS data has two types: 0.25° × 0.25° and 1° × 1°, and the time resolution has three types: three hours, one day, and one month. The precipitation and terrestrial water storage utilized in this study are from the GLDAS-2 data Catchment Land Surface Model, with a spatial resolution of 0.25° × 0.25° and a time resolution of one day, from 1 January 1960 to 30 December 2014. Moreover, it was necessary to employ GLDAS data here for two reasons: (1) due to the special geographical environment of the Tarim River Basin, the measured meteorological and hydrological stations in this area are scarce and spatial analysis cannot be effectively performed, while GLDAS data can account for the lack of observation data; (2) compared with other types of earth product data, GLDAS data have a longer time-span and relatively high temporal and spatial resolution, which can fulfill the needs of this study. Besides, many studies have affirmed that GLDAS data have better applicability in the Tarim River Basin [21].

### 2.2. Methodology

To quantitatively assess the response of hydrological drought to meteorological drought, we first used two indicators of SPI and SWSI to measure the spatiotemporal patterns of meteorological and hydrological droughts. Thereafter, the cross-wavelet method was employed to explore the temporal response of hydrological drought to meteorological drought via multi-time scales. Finally, the ensemble empirical mode decomposition (EEMD), time lag cross-correlation method, and correlation analysis method were utilized to explore the spatial response of hydrological drought to meteorological at different multi-time scales.

#### 2.2.1. Standardized Precipitation Index (SPI)

Numerous scholars have applied the Standardized Precipitation Index (SPI) [22], Z-index, Standardized Precipitation Evapotranspiration Index (SPEI) [23], and Palmer Drought Severity Index (PDSI) [24] to drought in the Tarim River Basin, and achieved good results [17,25,26,27,28], indicating that these measures have good applicability in describing droughts in Tarim River Basin. Standardized Precipitation Index (SPI) and Standardized Precipitation Evapotranspiration Index (SPEI) can measure changes in meteorological drought on multiple time scales, while Z index and PDSI can only measure changes in meteorological drought on a single time scale, and Palmer Drought Severity Index (PDSI) involves many meteorological parameters. However, this study mainly explores the response of hydrological drought to meteorological drought on multiple time scales in endorheic basins. Therefore, the Z index and PDSI are not suitable. Then, to identify which SPI or SPEI is more suitable to describe the meteorological drought in the Tarim River Basin, we use the scoring method to compare the meteorological drought levels calculated through SPI and SPEI with the actual meteorological drought levels. The index with a higher score is considered to be optimal. In the end, we found that the score of SPI is higher than that of SPEI. Compared to SPEI, SPI involves fewer parameters (mainly precipitation) and the data are easy to obtain. Due to the complex climate and geographical environment of the Tarim River Basin, the meteorological observation data here are seriously lacking, and the relevant reanalysis data also cannot meet the needs of multi-parameter indicators due to accuracy and other reasons. Therefore, SPI is chosen as an indicator to measure meteorological drought in the Tarim River Basin.

The SPI is frequently used for meteorological droughts because of its simple calculation process and easy data collection procedure [22,29]. The SPI was originally intended only for the calculation of monthly scale but was later improved by scholars [30]. Now, it can also be used in the calculation of daily scale. The general calculation process is as follows:

First, assuming that $p_{i,j}^{k}$ is the jth day of the ith year, the cumulative precipitation within k days, and the cumulative precipitation $p_{i,j}^{k}$ on a day of the ith year through the 90-day time scale k can be expressed as follows:

(1) $$\begin{array}{c} \{\begin{array}{c} p_{i,j}^{k}=\overset{90}{\underset{l=91-k+j}{\sum }}p_{i-1,l}+\overset{j}{\underset{l=1}{\sum }}p_{i,l},\;\;\;\;\left(\;j<k\right)\\ \;\;p_{i,j}^{k}=\overset{j}{\underset{l=j-k+1}{\sum }}p_{i,l},\;\;\;\;\;\;\;\;\;\;\;\;\;\;\;\;\;\;\;\;\;\;\;\;\;\;\;\left(j\geq k\right) \end{array}\;\; \end{array}$$

In practice, the distribution of precipitation does not conform to the normal distribution, which is usually described by the *Γ* distribution. Assuming that the precipitation in a certain period is *x*, the probability density function of its corresponding *Γ* distribution is:

(2) $$\begin{array}{c} f\left(x\right)=\frac{1}{\gamma^{\beta }\Gamma \left(\beta \right)}x^{\beta -1}e^{-\frac{x}{\gamma }}\;,\;\;\;\;\;\;\;\gamma >0,\beta >0,x>0\; \end{array}$$

where γ and β represent shape and scale parameters, respectively, and they can be obtained via the maximum likelihood method.

Afterward, on a given time scale, the cumulative probability of precipitation *x* can be expressed as:

(3) $$\begin{array}{c} \{\begin{array}{c} F\left(x\right)=\int_{0}^{x}f\left(x\right)dx=\int_{0}^{x}\frac{1}{\gamma^{\beta }\Gamma \left(\beta \right)}x^{\beta -1}e^{-\frac{x}{\gamma }}dx\;\;\left(x\neq 0\right)\\ F\left(x\right)=\frac{m}{n}\;\;\;\;\;\;\;\;\;\;\;\;\;\;\;\;\;\;\;\;\;\;\;\;\;\;\;\;\;\;\;\;\;\;\;\;\;\;\;\;\;\;\;\;\;\;\;\;\;\;\;\;\;\;\;\;\;\;\;\;\;\;\;\;\;\left(x=0\right) \end{array}\; \end{array}\;$$

In the above formula, *m* represents the number of samples with zero precipitation, and *n* represents the total number of samples. The following equation can be obtained by the standard normalizing formula:

(4) $$\begin{array}{c} \mathrm{SPI}=S\frac{t-\left(c_{2}t+c_{1}\right)t+c_{0}}{\left[d_{3}t+d_{2}\right)t+d_{1}]t+1.0}\;\; \end{array}\;t=\sqrt{2\ln\left(F\right)}$$

where *F* is the distribution probability of precipitation, *S* is the coefficient of the probability density function, while *F* > 0, *S* = 1, and *F* ≤ 0.5, *S* = −1; $c_{0}$ = 2.515517, $c_{1}$ = 0.802853, $c_{2}$ = 0.010328, $d_{1}$ = 1.432788, $d_{2}$ = 0.189269, $d_{3}$ = 0.001308 [19].

SPI can be split into different levels, and the drought level standard on the daily scale is similar to that on the monthly scale [31]. The specific standards are exhibited in Table 1.

#### 2.2.2. Standardized Terrestrial Water Storage Index (SWSI)

There are also different types of hydrological drought indicators, among which the Standardized Runoff Index (SRI), Streamflow Drought Index (SDI), Standardized Terrestrial Water Storage Index (SWSI), etc. are widely used. SRI and SWSI can measure hydrological drought changes on multiple time scales. However, since the SRI time series is calculated based on the observation data of a specific observation section or basin control station of a river, it describes the water surplus-deficit of a specific area or basin in a specific period as a whole, and Northwest China is located in mountainous areas, where hydrological observation are sparse and hydrological observation data are scarce, so it lacks spatial resolution and cannot portray water surplus-deficit on smaller spatial units. For an endorheic basin in the vast northwestern region of China, it is not objective to use the SRI of individual stations to describe its drought conditions as a whole. To overcome this shortcoming of SRI, SWSI is used in this study. In addition, the parameters involved in SWSI are mainly terrestrial water storage. Although the hydrological observation data in the Tarim River Basin are seriously lacking, different reanalysis data, such as GLDAS data and ECMWF data, all provide terrestrial water storage data, which can meet this article’s need and describe the hydrology of the endorheic basin in northwestern China from the perspective of time and space. Consequently, we selected the Standardized Terrestrial Water Storage Index (SWSI) [30] to measure the hydrological drought in the Tarim River Basin.

**Figure 2:**
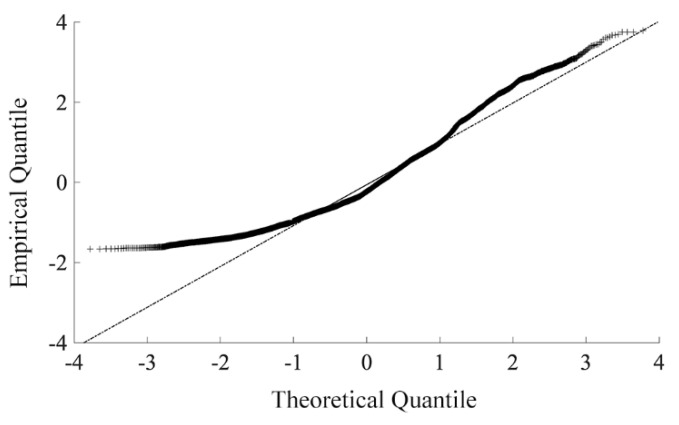
The scatter plot of empirical quantile and theoretical quantile of the daily terrestrial water storage.

The Standardized Terrestrial Water Storage Index (SWSI) determines whether hydrological drought occurs by explicating the profit and loss of terrestrial water storage [30]. We formulate the daily SWSI calculation based on the monthly SWSI calculation formula proposed by Wang et al. [30]. The specific process is as follows:

First, we compare the actual distribution of the terrestrial water storage with the standard normal distribution through the *Q-Q* chart. As evident from Figure 2, the scatter plot formed by the empirical quantile of the daily terrestrial water storage and the theoretical quantile of the standard normal distribution represents the same straight line. Therefore, we can treat the terrestrial water storage as the normal distribution. Then, the formula of *SWSI* can be outlined as follows:

(5) $$\begin{array}{c} SWSI_{i,\;\;j}=\frac{S_{i,j}-S_{j,mean}}{S_{j,std}}\; \end{array}$$

In the formula, $S_{i,j}$ signifies the terrestrial water storage on the *j*th day of the *i*th year, $S_{j,mean}$ denotes the mean value of the terrestrial water storage on a *j*th day, and $S_{j,std}$ represents the standard deviation of the terrestrial water storage on a *j*th day. The classification standard of the drought level is consistent with that of SPI (Table 1).

#### 2.2.3. Ensemble Empirical Mode Decomposition (EEMD)

The climate-hydrological system is a complex nonlinear system [32,33], which needs to be understood from a multi-scale and multi-level perspective. EEMD is a method that can effectively deal with nonlinear and non-stationary problems and appraise their internal mechanism [34]. This method has been widely used in meteorology and hydrology [35].

EEMD is the improvement in Empirical Mode Decomposition (EMD), which was first proposed by N. E. Huang in 1998 and was originally an adaptive time-frequency processing method. Although this method has been widely used, there are some vulnerabilities. The biggest problem is modal confusion, which causes the same component to be decomposed into different scales or allows the same scale to appear in different components [19]. EEMD can commendably address this problem, as it can adaptively decompose the time-frequency according to the changing characteristics of local time while eliminating the Fourier transform. The specific decomposition process is as follows:

(1) First, add the white noise of the specified amplitude to the original signal:

(6) $$\begin{array}{c} x_{i}\left(t\right)=x\left(t\right)+n_{i}\left(t\right)\; \end{array}$$

$x_{i}\left(t\right)$ exemplifies the signal after the addition of the white noise, x(t) specifies the original signal, and $n_{i}\left(t\right)$ represents the white noise.

(2) According to the principle of EMD decomposition, the signal after the addition of the white noise is decomposed to obtain the component IMF1.
(3) The same white noise is added to the signal after decomposing the component IMF1, and the new signal will continue to be decomposed according to the decomposition principle of EMD to acquire the component IMF2.
(4) The above steps will be repeated to derive multiple component IMFs, and the average of these components will then be used as the final result so that the white noise can be eliminated as exhibited in the following formula:

(7) $$\begin{array}{c} C_{j}\left(t\right)=\frac{1}{N}\overset{N}{\underset{i=1}{\sum }}C_{ij}\left(t\right)\; \end{array}$$

$C_{j}\left(t\right)$ displays the *j*th component IMF, *N* depicts the number of times the white noise is added, and $C_{ij}\left(t\right)$ presents the *j*th component after the addition of the *i*th white noise.

(5) Thereafter, different component IMFs and trend terms can be extracted and the signal reconstructed, as shown below:

(8) $$\begin{array}{c} x\left(t\right)=\overset{n}{\underset{j=1}{\sum }}C_{j}\left(t\right)+r_{n}\left(t\right)\; \end{array}$$

x(t) highlights the reconstructed signal, $C_{j}\left(t\right)$ symbolizes the different IMFs, and $r_{n}\left(t\right)$ represents the term of the trend.

#### 2.2.4. Cross Wavelet Transform (CWT)

The occurrence of meteorological drought and hydrological are not synchronized. In particular, hydrological drought lags behind meteorological drought, and the former has a response for the latter. This response can be quantified in a certain way so that we can understand meteorological and hydrological droughts more intuitively. CWT is an effective method, which combines continuous wavelet and cross-spectrum analysis, and can reveal the response relationship and relative phase relationship of two series at different time scales and investigate the correlation of two-time series through wavelet coherence [36].

Many studies have provided a detailed introduction of continuous wavelets [37,38]. Notably, the cross wavelet of two-time series $x_{n}$ and $y_{n}$ can be expressed as follows:

(9) $$\begin{array}{c} W^{XY}=W^{X}W^{Y*}\; \end{array}$$

where $W^{X}$ and $W^{Y*}$ are the cross wavelet transform coefficients and its complex conjugate, respectively. |$W^{XY}$| represents the energy of the cross wavelet. The cross-wavelet energy spectrum can reveal the common high-energy region of the two-time series and can reveal the relative phase relationship between the two-time series. This phase relationship can be characterized by the average phase angle, which provides the two lead-lag relationships of time series [36]. The average phase angle is described as follows:

(10) $$\begin{array}{c} \alpha_{m}=\arg\left(X,Y\right),\;X=\overset{n}{\underset{i=1}{\sum }}\cos\left(\alpha_{i}\right),\;Y=\overset{n}{\underset{i=1}{\sum }}\sin\left(\alpha_{i}\right)\;\; \end{array}$$

Furthermore, wavelet coherence can reveal the cross-wavelet transform’s coherence of the two-time series at different time scales, and is defined as follows:

(11) $$\begin{array}{c} R_{n}^{2}\left(s\right)=\frac{{\left|S\left(s^{-1}W_{n}^{XY}\left(s\right)\right)\right|}^{2}}{S\left(s^{-1}{\left|W_{n}^{X}\left(s\right)\right|}^{2}\right)\cdot S\left(s^{-1}{\left|W_{n}^{Y}\left(s\right)\right|}^{2}\right)}\; \end{array}$$

(12) $$\begin{array}{c} S\left(W\right)=S_{scale}\left(S_{time}\left(W_{n}\left(s\right)\right)\right) \end{array}$$

(13) $$\begin{array}{c} S_{time}\left(W\right){|}_{s}=\left(W_{n}\left(s\right)*c_{1}{}^{\frac{-t^{2}}{2s^{2}}}\right){|}_{s},\;S_{time}\left(W\right){|}_{s}=\left(W_{n}\left(s\right)*c_{2}\prod^{\;}\left(0.6s\right)\right){|}_{n}\; \end{array}$$

where *S* represents the smooth operator, s indicates the circular standard deviation, $s=\sqrt{-2\ln\left(\sqrt{x^{2}+\frac{y^{2}}{n}}\right)},\;S_{scale}$ outlines the smoothing along the wavelet scale axis, and $S_{time}$ implies smoothness in time. Furthermore, $c_{1}$ and $c_{2}$ are standardized constants, whereby ∏ represents a rectangular function, and 0.6 is the empirically determined scale decorrelation length of the Morlet wavelet. The significance test of wavelet coherence is generally tested via the Monte Carlo method.

#### 2.2.5. Time Lag Cross-Correlation

The relationship between various geographical elements is intricate but the simplest relationship is where a correlation exists between two geographical elements. Nevertheless, the correlation between the two factors may be synchronous, or there may be hysteresis [39]. For instance, the runoff in a month may correlate with the precipitation of the month, and that of the last month. Hence, based on the research topic of this paper, we introduce the time lag cross-correlation.

(14) $$\begin{array}{c} R_{xy}=\frac{\frac{1}{n-\tau }\sum_{i=1}^{n-\tau }\left(x_{i}-\bar{x_{i}}\right)\left(y_{i+\tau }-\bar{y_{i+\tau }}\right)}{\sqrt{\frac{1}{n-\tau }\sum_{i=1}^{n-\tau }\left(x_{i}-\bar{x_{i}}\right)}\sqrt{\frac{1}{n-\tau }\sum_{i=1}^{n-\tau }\left(y_{i+\tau }-\bar{y_{i+\tau }}\right)}}\; \end{array}$$

where $\bar{x_{i}}$ and $\bar{y_{i+\tau }}$ is the mean value, and $\bar{x_{i}}=\frac{1}{n-\tau }\overset{n-\tau }{\underset{i=1}{\sum }}x_{i}$, $\bar{y_{i+\tau }}=\frac{1}{n-\tau }\overset{n-\tau }{\underset{i=1}{\sum }}y_{i+\tau }$, τ is the time lag. $R_{xy}$ outlines the time lag cross correlation between two elements, and its value range is [−1,1]. If $R_{xy}$ > 0, a positive correlation between two elements is established, and if $R_{xy}$ < 0, a negative correlation between the two elements is determined. Moreover, if $R_{xy}$ = 0, no correlation between the two elements is identified. Notably, the closer the value of $R_{xy}$ is to 1, the greater the correlation between the two elements, and vice versa [39].

#### 2.2.6. Correlation Analysis

The correlation analysis method describes the closeness of the relationship between objective things and expresses it with appropriate statistical indicators, which are easy to understand, which has been widely used in various fields [40]. The calculation formula is as follows:

(15) $$\begin{array}{c} r=\frac{\sum_{i=1}^{n}\left(x_{i}-\bar{x}\right)\left(y_{i}-\bar{y}\right)}{\sqrt{\sum_{i=1}^{n}{\left(x_{i}-\bar{x}\right)}^{2}\sum_{i=1}^{n}{\left(y_{i}-\bar{y}\right)}^{2}}}\; \end{array}$$

In Equation (15), $x_{i}$ and $y_{i}$, respectively, represent the values of two variables; x and y are the average values of the two variables, respectively; r is the correlation coefficient, and when r > 0, the two variables are positively correlated, while when r < 0, the two variables are negatively correlated. A larger | r| indicates a stronger correlation.

## 3. Results

### 3.1. The Spatiotemporal Variations of Meteorological Drought and Hydrological Drought

**Figure 3:**
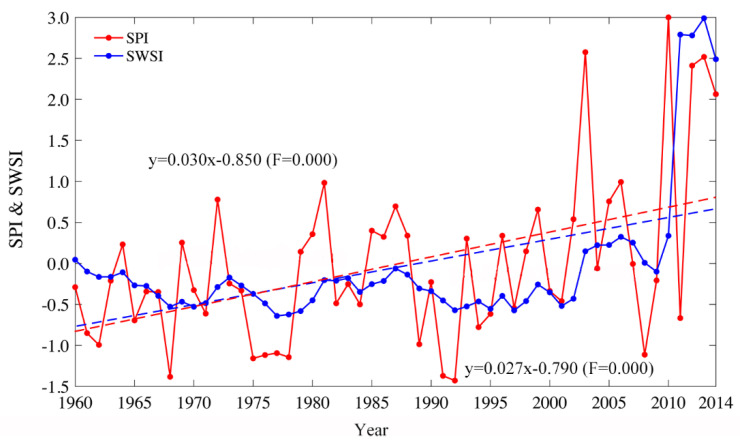
The variations of annual SPI and annual SWSI during the period of 1960 to 2014.

SPI and SWSI are utilized to measure the meteorological drought and hydrological drought, respectively. Initially, we calculated the SPI and SWSI on a 12-month time scale and adopted the value of the 12th month as the annual value of the SPI and SWSI. As is evident from Figure 3, it shows the variations in the annual value of the SPI and SWSI, and the SPI and SWSI all suggest a significantly increasing trend, verifying that both meteorological and hydrological droughts have gradually weakened from 1960 to 2014 in the Tarim River Basin. The minimum value of SPI is between −1.0 and −1.5, indicating that the average meteorological drought in the entire basin is moderate drought, while the minimum value of SWSI is between −0.5 and −1, indicating that the average intensity of the hydrological drought is deemed as light. Besides, the increasing trend in SPI is more obvious than SWSI, and the variation range of SPI is also larger than that of SWSI. 

Interestingly, meteorological drought is mainly affected by climate change and hydrological drought is influenced by both climate change and human activities. Notably, human activities, such as hydrological regulations, have a regulating effect on hydrological drought. Further, the variation trend of SPI was relatively stable before 2002, but increased rapidly after 2002, while the variation trend of SWSI was relatively stable before 2010, and increased rapidly after 2010. The variations in SPI and SWSI were also not consistent. The result of Zhang [41] proved that annual precipitation has rapidly increased since 2010 and, in turn, caused an increase in SPI and SWSI variations.

**Figure 4:**
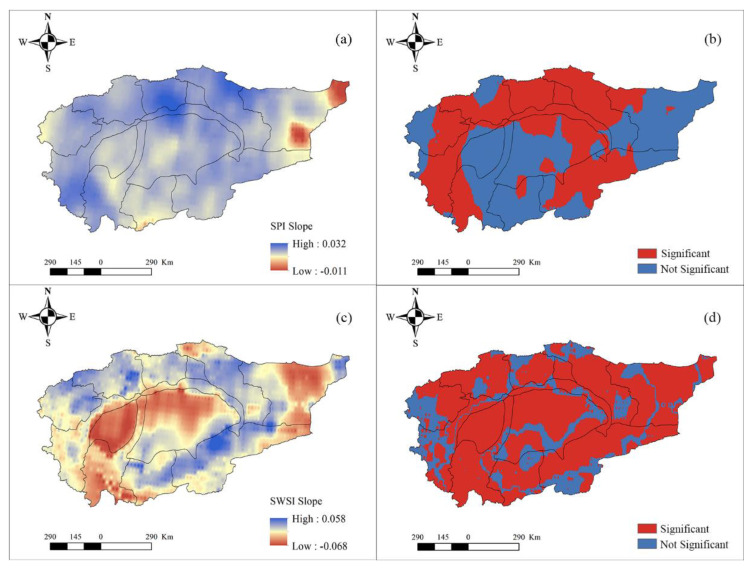
The spatial pattern of the SPI slope (**a**) and its significance (**b**) and SWSI slope (**c**) and its significance (**d**) during 1960–2014.

The spatial patterns of meteorological and hydrological droughts are also explored in Figure 4. From the spatial patterns of SPI (Figure 4a) and SWSI (Figure 4c), we can see that both spatial patterns are quite different. Figure 4a demonstrates that SPI has a significantly increasing trend in large areas, among which the increasing trend of SPI in the Taklimakan desert area and the Keliya river basin are not obvious. This means that the meteorological drought in most parts of the Tarim River Basin has gradually weakened. Previous studies have highlighted that the precipitation in the Tarim River Basin shows an increasing trend, and the precipitation in mountainous areas increased more than that in plain areas [19]. Therefore, meteorological drought in this basin shows a weak trend. Moreover, there is only a small area in the east of the Tarim River Basin that denotes a non-significant decreasing trend (Figure 4b), implying that the meteorological drought in these regions has gradually increased. As for the SWSI, the Hotan river basin, Taklimakan desert area, upper reaches of the mainstream basin, mountainous areas of the Kaikong river basin, and Qarqan river basin signify a significant decreasing trend (Figure 4c,d), confirming that hydrological drought in these regions has gradually increased. Contrarily, hydrological drought in other regions shows a decreasing trend. Thus, the spatial pattern of hydrological drought is more complex than meteorological drought because of the dual effect of climate change and human activities.

### 3.2. The Temporal Response of Hydrological Drought to Meteorological Drought

After a period of unalleviated meteorological drought, and because of the continuous increase in evaporation, it is possible to supplement the incidence of hydrological drought. Similarly, the intensity or duration of the hydrological drought is closely related to the meteorological drought [42]. Therefore, we first evaluate the relationship between SPI and SWSI through a simple correlation that could accurately distinguish the relationship between the two variables [43]. Table 2 illustrates that the correlation coefficient between them is 0.554 and the *p*-value is 0.000, implying a significant correlation between meteorological and hydrological droughts, which is consistent with previous studies [10,11,41]. Further, there are different correlations between SPI and SWSI in the four seasons, and the correlation between the two is the strongest in spring and weakest during winter.

**Figure 5:**
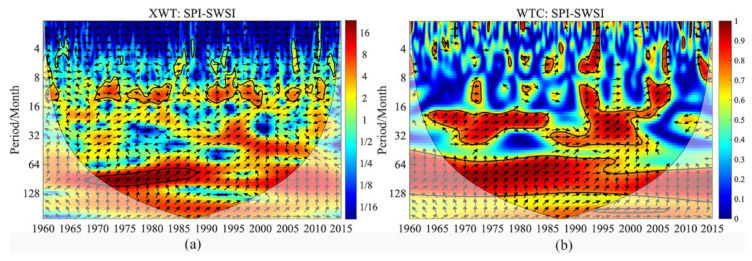
The cross wavelet transform XWT (**a**) and the wavelet coherence WTC (**b**) between SPI and SWSI.

Afterward, we studied the correlation between SPI and SWSI on the multi-time scale through the cross wavelet transform method (Figure 5). Based on this correlation, we can calculate the lag time of hydrological drought to meteorological drought via cross-phase angle and period.

Figure 5a is the cross wavelet transform XWT, and Figure 5b is the wavelet coherence WTC between SPI and SWSI. The shades of colors indicate the level of energy density, where red indicates high energy density and blue indicates low energy density. The closed thick black solid line indicates that the 5% significance test is passed. The thin black solid line of the cone is the cone of wavelet influence (COI), which signifies the area where the edge effect of the wavelet transform data has a greater influence. The direction of the arrows specifies the relative phase difference between meteorological and hydrological droughts, where the horizontal arrow to the right highlights the same phase between the meteorological and hydrological droughts, and the horizontal arrow to the left indicates the opposite phase between the two. The vertical upward arrow asserts that the meteorological drought occurred one quarter cycle earlier than the hydrological drought, and the vertical downward arrow confirms that the hydrological drought commenced one quarter cycle earlier than the meteorological drought.

Figure 5a exhibits a resonance period between the hydrological drought and the meteorological drought on the 10-month scale from 1970–1974, 1975–1983, and 1990–2000, and they all pass the significance test, indicating that the two have significant resonance on the 10-month time scale. The direction of the arrow denotes that the resonance of the hydrological and meteorological droughts on the 10-month scale is not very stable, and the average phase angle is 0.179 ± 1.731 radians, that is, the average of hydrological drought lags behind meteorological drought by 12 days. Additionally, there is a main resonance period between hydrological and meteorological droughts on the 8-year scale from 1970 to 1987. The average phase angle of the two is 1.163 ± 0.203 radians. Hence, the average of hydrological drought lags behind meteorological drought by 556 days. It can be seen that the response of the hydrological drought to the meteorological drought is different on different time scales. Further, Figure 5b presents the wavelet coherence of SPI and SWSI, which can make up for the deficiency of the cross-wavelet transform of the correlation between the two-time series concerning the low energy region. SPI and SWSI highlight the intermittent positive coherence on a short time scale of 4–8 months, and the coherence is relatively weak. Therefore, on a short time scale, the resonance cohesion of the two is not strong. On the 16–32-month scale, 8–32-month scale, and 60–128-month scale periods, hydrological and meteorological droughts showed relatively stable positive coherence, indicating that hydrological drought is greatly impacted by meteorological drought on a long time scale.

### 3.3. The Spatial Response of Hydrological Drought to Meteorological Drought

Primarily, the occurrence of hydrological and meteorological droughts is not synchronized, and hydrological drought will lag behind meteorological drought for a long time. Hence, how does this hysteresis behave spatially? We first used the lag correlation method to explore the spatial lag relationship between the hydrological and meteorological droughts in the Tarim River Basin. Moreover, the order corresponding to the maximum and significant correlation coefficient between the SPI and SWSI is used as the lag time of the hydrological drought in response to meteorological drought [10].

**Figure 6:**
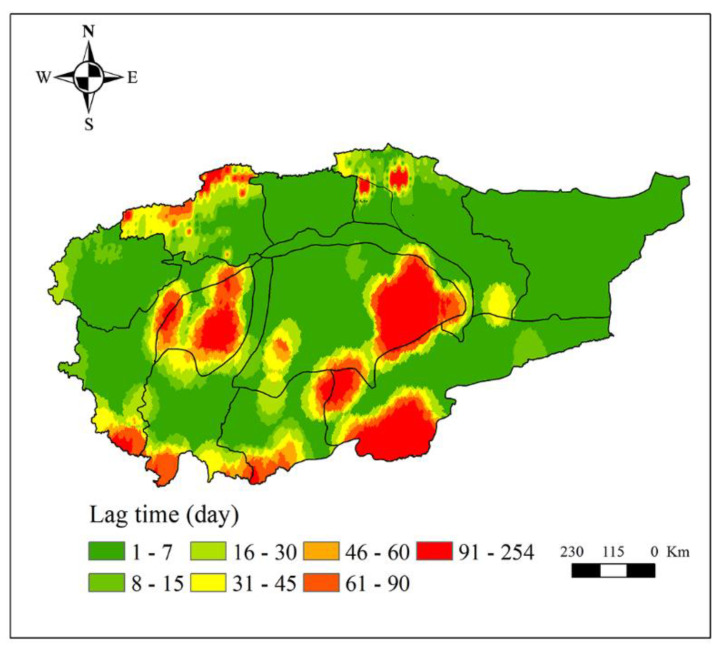
The lag time of hydrological drought to meteorological drought.

From Figure 6, we can see that the lag time of hydrological drought to meteorological drought in the Aksu, Qarqan, Yerkand, Hotan, Keliya, and Kashgar river basins has obvious hierarchical characteristics. From plain to mountainous areas, the lag time of hydrological drought in response to meteorological drought is gradually increasing. In the mountainous areas of each sub-basin and the Taklimakan desert, the lag time is long (i.e., up to one year), while that in plain areas is relatively short (i.e., mostly within 7 days). Among them, the spatial patterns of the response of hydrological drought to meteorological drought are similar to the Aksu and Kaikong river basins in the north of the Tarim River Basin. In these two basins, hydrological drought in the plains is affected by both climatic factors and human activities. When meteorological drought occurs, hydrological drought will soon occur due to the interference of human activities. The closer the region is to the mountainous area, the lower the impact of human activities. Under the effect of a single factor, the lag time of hydrological drought to meteorological drought will be longer. Therefore, the lag time in the mountainous area is longer than that in the plain area. Interestingly, the spatial patterns are similar in the Yerkand, Hotan, and Qarqan river basins in the south of the Tarim River Basin. Moreover, the lag time in the mountains of these areas is significantly longer than the lag time in the mountains of the Aksu and Kaikong river basins. The difference is that the former set of river basins has a distribution of a large number of glaciers on their mountains. When the meteorological drought occurs, due to the regulation of glacial meltwater, hydrological drought will not occur in a short time, as in the latter two river basins. Although the Kashgar river basin also has a large area of glaciers, the lag time of hydrological drought to meteorological drought in the mountainous area is much shorter than that of the Yarkand and Hotan river basins (i.e., 30 days). These differences need further research. 

**Figure 7:**
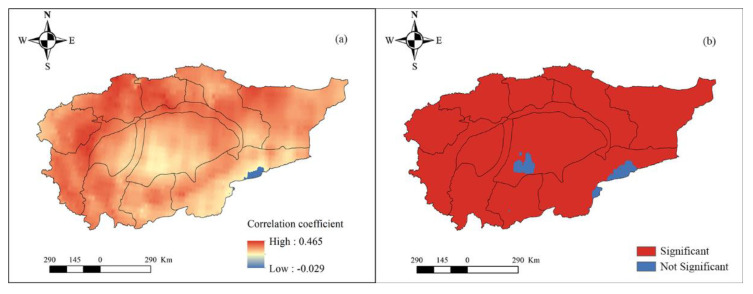
The correlation between SPI and SWSI on a monthly scale (**a**) and its significance (**b**).

**Figure 8:**
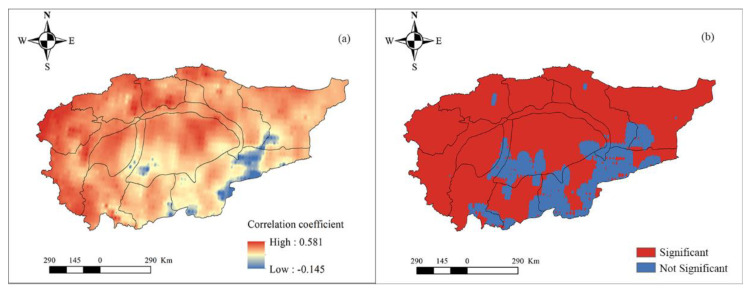
The correlation between SPI and SWSI on an inter-annual scale (**a**) and its significance (**b**).

**Figure 9:**
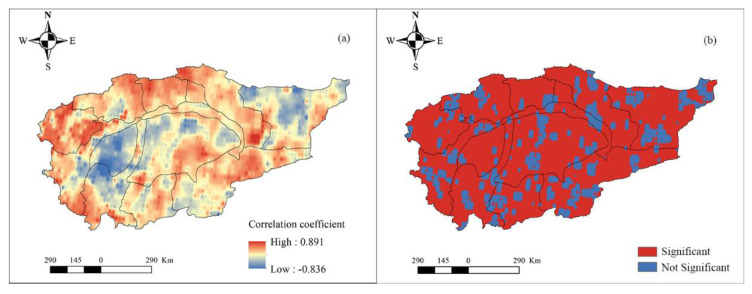
The correlation between SPI and SWSI on an inter-decadal scale (**a**) and its significance (**b**).

We also discuss the spatial response of hydrological drought to meteorological drought from the perspectives of different time scales. First, the SPI and SWSI of each grid were calculated. Next, the EEMD decomposition of SPI and SWSI of each grid was performed, and the components of SPI and SWSI on the month scale, inter-annual scale, and inter-decadal scale were calculated, respectively. Lastly, the correlation between the SPI and SWSI on different time scales was separately obtained. On the monthly time scale (refer to Figure 7), the correlation between hydrological and meteorological droughts is positive (i.e., Figure 7a) and passes the significance test (i.e., Figure 7b). Only the sporadic area reports a negative correlation in the Qarqan river basin and fails to pass the significance test. The correlation between hydrological and meteorological drought is relatively high in the Aksu, Weigan, Yarkant, and Kashgar river basins, but the correlation is relatively low in other basins, especially the Taklimakan desert. On the interannual time scale (refer to Figure 8), the spatial pattern is similar to the spatial pattern on the monthly time scale, and the correlation between hydrological and meteorological droughts is positive (Figure 8a) and passes the significance test. Furthermore, some sporadic area depicts a negative correlation in the Qarqan and Keliya river basins, and desert area; those areas all fail to pass the significance test (i.e., Figure 8b). The correlation between the mountainous areas of Aksu, Weigan, Kaikong, Yarkand, Hotan, and Kashgar river basins was relatively high. On the inter-decadal time scale (refer to Figure 9), the spatial pattern is complex, and the correlation between hydrological and meteorological droughts in mountainous areas is positive while the correlation between them is negative in the Taklimakan and Kumtag deserts, and some plain areas. From Figure 9a, it is evident that the positive and negative correlations between meteorological and hydrological droughts are staggered, and the correlation between them is higher than the correlation on the monthly and inter-annual time scales. Hence, hydrological drought is more affected by meteorological drought on a long time scale. 

## 4. Discussion

The multi-time scale response of hydrological drought to meteorological drought is a relatively unexplored problem in the Tarim River Basin due to the lack of measured meteorological and hydrological data. To solve this problem, the cross wavelet transform and the EEMD method is used to explore the temporal and spatial response of hydrological drought to meteorological drought through the reanalysis data. In this study, SPI and SWSI are used to evaluate the temporal and spatial variation of meteorological and hydrological drought, respectively. We found that they all showed a weakening trend from 1960 to 2014, but there was a large difference in space. Thereafter, we explored the temporal response of hydrological drought to meteorological drought on the multi-time scale. The result showed that meteorological and hydrological drought have significant resonance periods on the 10-month time scale and the 8-year time scale. The spatial response of hydrological drought to meteorological drought on the multi-time scale was also analyzed, and the former generally lags behind the latter by 7 days in plain areas, while the lag time of hydrological drought to meteorological drought can last as long as several months or even a year in mountainous areas. Besides, the two showed a significant positive correlation in the entire basin on the monthly and inter-annual time scales. On the inter-decadal time scale, the spatial pattern of the correlation between the two is more complicated, and deserts had a large area of negative correlation.

Our findings indicate that the meteorological and hydrological droughts in the Tarim River Basin all show a tendency to weaken from 1960 to 2014, which is consistent with some studies [10,30]. Alternatively, the variation trend of hydrological drought is more obvious than the variation trend of meteorological drought before 1980, while the opposite is true after 1980. In the past century, the global average temperature has gradually increased, and the same is true for the Tarim River Basin [44]. This increase in temperature caused the melting of glaciers and snow and increased terrestrial water storage, which gradually weakens the hydrological drought. Chen et al. [45] proved that both temperature and precipitation in the Tarim River Basin underwent abrupt changes in the 1980s, and the increasing trend of precipitation was more obvious than that of temperature. Therefore, the weakening trend of the hydrological drought was lower than that of the meteorological drought after the 1980s.

There are different types of drought, such as meteorological, hydrological, agricultural, and socio-economic droughts, and there are differences and connections between these different types. Generally, meteorological drought is the precursor of other types of drought and other types of droughts respond to meteorological drought. Although previous studies had explored this issue [46,47,48,49], wherein most used the simple correlation method to analyze the relationship between hydrological and meteorological droughts. For example, Barker et al. [46] investigated the relationship between meteorological drought and hydrological drought by using the Standardized Precipitation Index (SPI) and Standardized Streamflow Index (SSI) in the UK and pointed out that SPI has the strongest correlation with one month SSI. Wu et al. [47] used the Standardized Streamflow Index (SSI) and Standardized Precipitation Index (SPI) in different time scales (1, 3, 6, 12, and 24 months) to compare the relationship between hydrological drought and meteorological drought before and after the construction of the reservoir. Compared with the pre-reservoir period (1960–1972), reservoir operations in the post-reservoir period (1974–2015) changed the linear correlation between hydrological drought and meteorological drought. Li et al. [48] found that there is a strong correlation between the Standardized Precipitation Index (SPI) and Standardized Runoff Index (SRI) in all three subareas of the Huai River Basin, particularly at the 6 month timescale. The increasing influences of human activities (e.g., regulation of water conservancy facilities) from upstream to downstream lead to a weaker correlation between SPI and SRI. The formation of the two types of drought is complex and diverse; thus, this study explored the response of hydrological drought to meteorological drought from the perspective of multi-time scales. The results show that, on a short time scale, the resonance between the two is complex and has a short duration, while on a long time scale, the two have significant and stable resonance and signify a long duration. Further, on a short time scale, such as a monthly time scale, hydrological drought is not only affected by meteorological drought but also affected by human activities to a greater extent. In agriculture, groundwater or river and lake water are directly extracted to irrigate crops. Part of it is absorbed by crops and soil and the rest transpires into the air to participate in the next water cycle. Only a small part of it may infiltrate the soil to replenish the groundwater. Consequently, the water level of surface water and groundwater will decrease. Notably, most of the freshwater used in the industrial production process comes from surface water and groundwater, which can be used as raw material water, boiler water, and cooling water [50]. Nonetheless, industrial water faces the problem of low water efficiency. The repetition rate of industrial water in small towns is only 52% in China [51], which requires the continuous extraction of surface water and groundwater to satisfy industrial water demand. On the other hand, industrial water also discharges a large amount of sewage and wastewater, causing serious pollution problems. In terms of social life, urban greening, people’s daily washing, and water-related entertainment, projects are inseparable from water. All of these may lead to hydrological drought. Therefore, the resonance between the hydrological drought and meteorological drought is complex. Comparatively, on a long time scale, such as the inter-decadal time scale, hydrological drought is mainly affected by meteorological drought. Compared with previous studies, our research comprehensively analyzed the response of hydrological drought to meteorological drought, which enables us to have a deeper understanding of drought-related issues.

Due to the lack of measured meteorological and hydrological data, there is a scarcity of research on the spatial response of hydrological drought to meteorological drought in the Tarim River Basin. This study makes up for the deficiencies in previous studies, and it is revealed that hydrological drought in plain areas generally lags behind meteorological droughts by 7 days, while in mountainous areas, it can be as high as several months or even a year, reflecting the difference response sensitivity of hydrological drought to meteorological drought in regions similar in climate but differing in catchment characteristics. The reason for this difference is in addition to the aforementioned climate and human activities, many studies have confirmed that catchment attributes (e.g., water storage, groundwater, vegetation, altitude, soil aquifer, and geology) have an important impact on the response time of hydrological drought to meteorological drought [52,53,54]. The mountainous area of the Tarim River Basin has a high altitude and is accompanied by a large amount of glacier snow. The glacier snow melt water has a regulating effect on hydrological drought in the mountainous area and can alleviate the occurrence of hydrological drought. When glacier snow melt flows into the plain area, due to the influence of evaporation and infiltration, the mitigation effect of the hydrological drought in the plain area is weaker than that of the mountainous area. As a result, when meteorological drought occurs, the response time of hydrological drought is different. Besides, vegetation has a significant role as the soil structure is directly related to the hydrological cycle processes (e.g., the runoff occurred during a heavy precipitation event or the water drainage from the aquifers to the watersheds). Additionally, natural vegetation consumes water for respiration processes, and the vegetation coverage in the mountains of the Tarim River Basin is significantly higher than that in the plains, which is also the main factor for the difference in drought response time. Furthermore, the soil moisture in mountainous areas is also significantly higher than that in plain areas, whether it is within a thickness of 0–10 cm, 10–40 cm, or 40–100 cm [55]. Higher soil moisture will also alleviate the occurrence of hydrological drought. Studies have also analyzed the possible impact of large-scale ocean-atmospheric factors (such as the El Niño Southern Oscillation) on the response time of hydrological drought to meteorological drought [11,56]. Although we do not quantitatively discuss the impacts of these additional factors on the response time of hydrological drought to meteorological drought in our paper, our findings suggest that catchment differences should be considered when warning based on hydrological drought.

The study also found that, as the time scale becomes longer, the correlation between meteorological drought and hydrological drought becomes greater and the correlation between meteorological and hydrological droughts in mountainous areas is higher than that of the plain areas. The population of the Tarim River Basin is mainly distributed along these basins wherein the human activities are mainly concentrated in the plains, and the mountain areas remain less affected. Therefore, the correlation between meteorological and hydrological droughts in mountainous areas is higher than that of the plain areas. Ma et al. [10] state that the duration by which the hydrological drought lags behind the meteorological drought varies with seasons. Similarly, Barker et al. [46] indicated that hydrological drought lagging behind meteorological drought have different times of onset in different regions, ranging from 1 month to 12 months. Notably, they only use the monthly scale drought index to examine the lag time of hydrological drought in response to meteorological drought. Accordingly, they concluded that hydrological drought lags behind meteorological drought for at least one month. In fact, hydrological drought may lag behind meteorological drought for a few days, so there may be errors in their results. Compared with previous studies, our study uses the daily-scale drought index to explore the response of hydrological drought to meteorological drought, which improves the accuracy of the results.

This study investigated the spatiotemporal response characteristics of hydrological drought to meteorological drought from the multi-time scale. Building on this, the response mechanism of hydrological drought to meteorological drought warrants further research. 

## 5. Conclusions

This study first assessed the spatiotemporal variation of meteorological drought and hydrological drought from 1960 to 2014 using the SPI and SWSI of the Tarim River Basin. We explored the temporal response of hydrological drought to meteorological drought on the multi-time scale by using the cross-wavelet transform and EEMD methods. Finally, the spatial response of hydrological drought to meteorological drought on the multi-time scale was appraised. Summarizing this study, the main conclusions are as follows:

1. Meteorological drought and hydrological drought all showed a trend of getting weak from 1960 to 2014, and the variation trend of hydrological drought is more obvious than that of meteorological drought before 1980, while the opposite is true after 1980. In space, meteorological drought depicted an overall weakening trend, while hydrological drought showed a weakening trend in plain areas and an increasing trend in desert areas; the change was not obvious in mountainous areas.
2. Meteorological and hydrological drought have significant resonance periods on the 10-month time scale and the 8-year time scale. The correlation between meteorological drought and hydrological drought is not stable in the resonance period at the short time scale, while it is stable at the long time scale.
3. Hydrological drought develops in different time scales and spatial distributions in the study region. Additionally, it is particularly important that different responses develop in the lowland and mountainous parts, suggesting that we need to approach the two topographic situations differently.

The occurrence of drought is an extreme reflection of a series of water cycle elements. From meteorological drought to hydrological drought, it is an inherently related process. Through the correlation between different regions and different drought types, the local government should formulate some drought prevention measures according to local conditions. For example, hydrological drought in the plains of the Tarim River Basin generally lags behind meteorological drought within 7 days. It can be considered that meteorological drought has a greater impact on hydrological drought. Therefore, the surface water should be recharged in a short time after the occurrence of meteorological droughts, such as releasing water from reservoirs to alleviate the downstream drought, to avoid and prevent various losses caused by meteorological drought. According to the changing trends of SPI and SRI, these two types of drought indicators show an increasing trend in a large area in my country’s Tarim River Basin. Therefore, it is possible to provide certain suggestions to drought control personnel, local governments or researchers, and to make future predictions on the drought conditions in the northwest endorheic basin. At the same time, we can encourage everyone to carry out afforestation activities in the northwest endorheic basin, improve the climate and environment in the western region, save and protect water resources, and prevent drought disasters.

## Figures and Tables

**Table 1:** The Drought Level Standard.

Category	SPI Value
Normal	−0.5 < SPI
Mild drought	−1 < SPI ≤ −0.5
Moderate drought	−1.5 < SPI ≤ −1
Severe drought	−2 < SPI ≤ −1.5
Extreme drought	−2 ≥ SPI

**Table 2:** The simple correlation of SPI and SWSI.

	Correlation Coefficient	*p*-Value
Year	0.554 ***	0.000
Spring	0.456 ***	0.000
Summer	0.289 ***	0.000
Autumn	0.232 ***	0.003
Winter	0.197 **	0.012

Note: ***, ** denote the passing of the significance test at 1% and 5%, respectively. Spring refers to March, April, and May; summer refers to June, July, and August; autumn refers to September, October, and November; winter refers to December, January, and February. The seasonal values of SPI/SWSI are calculated by considering the whole series and then categorizing them according to season.

## Data Availability

All data, models, and code generated or used during the study appear in the submitted article.
